# The Predicting Power of Cognitive Fluency for the Development of Utterance Fluency in Simultaneous Interpreting

**DOI:** 10.3389/fpsyg.2020.01864

**Published:** 2020-08-04

**Authors:** Shuxian Song, Dechao Li

**Affiliations:** ^1^School of Translation Studies, Qufu Normal University, Rizhao, China; ^2^Department of Chinese and Bilingual Studies, The Hong Kong Polytechnic University, Kowloon, Hong Kong

**Keywords:** simultaneous interpreting, cognitive fluency, utterance fluency, attention control, lexical access, working memory capacity

## Abstract

Although simultaneous interpreting (SI) is generally recognized as a highly demanding cognitive activity in nature, the role of cognitive processes in SI fluency is yet to be determined. While utterance fluency refers to the set of objectively determined oral features of utterances, cognitive fluency means the speaker’s efficient mobilization and integration of underlying cognitive processes responsible for utterance production. An investigation into the relationship of the two dimensions of fluency helps to reveal the cognitive bases of interpreting. This study explores the predicting power of cognitive fluency in the utterance fluency development of L2 (English)–L1 (Chinese) SI output of trainee interpreters. Cognitive fluency was operationalized as measures of lexical access, linguistic attention control, and working memory capacity. Measures of utterance fluency were obtained through simulated SI tasks under conditions of low and high input rates. Twenty-eight trainees interpreted two speeches, one with a high input rate and the other with a low input rate, at the beginning and end of an SI training period of 13 weeks. A bilingual corpus of the participants’ SI output was built, and indicators of SI utterance fluency were annotated systematically. Utterance fluency was indexed by the speech rate, mean length of run, phonation time ratio, mean number of silent pauses, and mean number of disfluencies. Results of analyses indicated that (1) the predicting power of cognitive fluency for SI utterance fluency development was only shown under high cognitive load over a training period of 13 weeks; (2) predictors for the development of SI utterance fluency tended to be the efficiency of cognitive processes involved in the target language production stage; and (3) the inclusion of measures of working memory capacity significantly increased the predicting power of cognitive fluency for SI utterance fluency development. This study for the first time provides evidence for the role of cognitive fluency in trainee interpreters’ SI utterance fluency development, having implications for the theoretical framework of cognitive fluency and the information processing mechanism in interpreting process, as well as for interpreter aptitude tests and interpreting pedagogy.

## Introduction

Simultaneous interpreting (SI) is a complex bilingual activity. It involves the comprehension of message in one language and the immediate verbal rendition of it into another language while the interpreter keeps listening to the incoming information (Liu et al., 2004; Injoque-Ricle et al., 2015). Simultaneous interpreting involves concurrent listening and speaking for a substantial percentage of the speech time, and it requires flexible and efficient online processing of cognitive resources in order to produce full and fluent delivery (Shlesinger, 2003). The efficiency of the interpreter’s cognitive processes is in particular important for successful SI due to the severe time pressure and high cognitive demand.

Fluency, being one of the most important quality criteria in interpreting (Liu et al., 2008), is important in the overall interpreting evaluation. It is an important feature of successful interpreting (Mead, 2000). Fluency is defined by Lennon (2000, p. 26) as “the rapid, smooth, accurate, lucid, and efficient translation of thought or communicative intention into language under the temporal constraints of online processing.” A crucial factor in fluency is the efficiency of the speaker’s cognitive processes underlying speech production. Inefficient linking of words to meanings might slow down the overall processing and create overload problems in short-term memory (Goldman-Eisler, 1968). Previous studies on fluency in interpreting are mostly descriptive. These studies mainly focus on disfluencies, which signify difficulties and uncertainties encountered in the cognitive processes of interpreting (Gósy, 2007; Bakti, 2009). An investigation into fluency will enhance our understanding of the information processing mechanism of SI and help to understand the cognitive bases of interpreting.

Fluency is a multidimensional concept. The framework of Segalowitz (2010) illustrates three domains of fluency, that is, cognitive fluency, utterance fluency, and perceived fluency, and their relationships. Cognitive fluency refers to fluid operation of the speaker to mobilize and integrate the underlying cognitive processes responsible for utterances production. Cognitive processes involved in cognitive fluency include, among others, the speed and efficiency of lexical access, linguistic attention control, and operations in working memory (Segalowitz and Freed, 2004; Segalowitz and Frenkiel-Fishman, 2005; Segalowitz, 2010, 2016; De Jong et al., 2013; Lim and Godfroid, 2014). Utterance fluency reflects the impact of cognitive processes and refers to the set of objectively determined oral features of utterances, representing the characteristics a speech sample possesses, for example, the temporal, hesitation, and repair features; perceived fluency refers to listeners’ inferences about a speaker’s cognitive fluency based on their perception of utterance fluency in the speech output (Segalowitz, 2010). The underlying cognitive system carries out functions of utterance planning and assembling. With the integration of these functions, utterances are executed with the desired features of oral production. The domain of cognitive fluency is the operation of these planning and assembling functions and their integration and execution (ibid.). This study aims to examine the relationship of cognitive fluency and utterance fluency development in the SI output of trainee interpreters under conditions of low and high cognitive load.

The current study included lexical access, linguistic attention control and working memory capacity in its operationalization of the cognitive fluency constructs. Lexical access and linguistic attention control were aspects that studies on cognitive fluency in language learning usually explored. Although it was stated that cognitive fluency involved operations of working memory (De Jong et al., 2013), previous studies in second language learning seldom included it in their exploration into cognitive fluency. Working memory capacity was included in this study as it is important for interpreting (Macnamara and Conway, 2016). It would also verify the effectiveness of including working memory capacity as a construct of cognitive fluency in interpreting research. In the field of interpreting studies, cognitive factors have been found important for interpreting performance (Christoffels et al., 2003; Injoque-Ricle et al., 2015; Macnamara and Conway, 2016; Lin et al., 2018). Previous interpreting studies on the relationship of cognitive fluency related aspects and interpreting performance, in particular, lexical access and retrieval, cognitive control, and working memory capacity, were introduced in the following.

Lexical access is the access of lexical entries from the mental lexicon, containing the stored information of the forms and meanings of words, in which basic sound-meaning connections of a language are activated (Field, 2004). It is a fundamental skill required for most aspects of language performance. The efficiency of the access of words or meaning and the translation equivalents are crucial for the task of interpreting. A highly related process of lexical access is lexical retrieval, with many components interchangeable with those of lexical access, although the order in which the process components are activated is reversed (Levelt et al., 1999; Snellings et al., 2002). Some studies offered empirical support for the links between lexical access or retrieval and interpreting. Interpreting training and experience was found to develop a set of cognitive skills including faster access to lexical and semantic information and larger working memory capacity in the study of Bajo et al. (2000). Their data showed that interpreters had advantage in the access to semantic and lexical information, whereas bilinguals did not show this superiority. The study of Christoffels et al. (2003) revealed a correlation between interpreting performance and lexical retrieval, as measured through word translation and picture naming tasks. However, Cai et al. (2015) failed to find a significant influence of lexical retrieval, elicited from a translation recognition task, on interpreting performance of student interpreters in their exploration into factors contributing to individual differences in the development of consecutive interpreting competence. Admittedly, differences in the tasks of elicitation, modes of interpreting, and profiles of participants in these studies might contribute to their divergent findings. This proves the needs for further well-designed experimental studies on the contribution of lexical access to interpreting performance.

The nature of simultaneity of comprehension and production of SI means that the control of attention is important. Linguistic attention, also called language-directed attention, forms an essential component of cognitive fluency (Talmy, 2008; Segalowitz, 2010). For linguistic attention control, “the control of attention originates from the linguistic message itself and is directed back to the mental representation that is associated with the meaning of the message” (Segalowitz, 2010, p. 95). The control of attention can be reflected by a person’s ability to “shift focus of attention from one language-based attention-directing function to another,” and a superior ability to make these shifts rapidly is assumed to indicate better control of language-directed attention (Segalowitz and Frenkiel-Fishman, 2005, p. 646). Previous interpreting studies seldom involved linguistic attention control, but relevant studies have investigated the links between interpreting performance and cognitive control, which consists of three functions, that is, inhibition, shifting, and updating (Miyake et al., 2000). The construct of linguistic attention control taps into the shifting function in the current research. These studies have implications for the current investigation into linguistic attention control of interpreters. Shifting and updating functions were found to reflect cognitive abilities that were important for interpreting in the research of Timarová et al. (2014). However, some studies found negative relationship between domain-general cognitive control and interpreting performance. For instance, Babcock and Vallesi (2017) showed that interpreters did not continue to garner benefits from bilingualism, although had a verbal and spatial memory advantage. Several empirical studies support that interpreting experience contributes to the enhancement of one or more functions of cognitive control (Dong and Xie, 2014; Becker et al., 2016; Dong and Liu, 2016; Dong et al., 2018), including but not limited to the updating skills (Morales et al., 2015) and attention processing and monitoring (Dong and Zhong, 2017). Differences in the participants profile and the choice of cognitive tasks might explain the discrepancy in findings. The disparate findings listed above entail the need for further exploration into links between different functions of cognitive control and interpreting performance.

Working memory refers to a cognitive system that can temporarily store and process information, which retains information in an accessible state suitable for carrying out tasks with a mental component and is essential for complex cognitive tasks and language processing (Baddeley and Hitch, 1974; Cowan, 1999; Caplan et al., 2007). Working memory has a limited capacity and requires “simultaneous storage and processing of information” (Baddeley, 1992, p. 556), playing an essential role in cognitive processing tasks including language comprehension and production. Highly demanding on working memory, interpreting is regarded as a process of maintaining equilibrium between different cognitive demands (Chernov, 2004). Previous studies have shown that working memory capacity correlates with both L1 and L2 utterance fluency (Daneman, 1991; Fortkamp and Bergsleithner, 2007). Despite the fact that the exact role of working memory capacity in interpreting has not reached a consensus, the importance of working memory in SI is generally acknowledged (Injoque-Ricle et al., 2015; Macnamara and Conway, 2016; Yenkimaleki and van Heuven, 2017). Some studies have provided support for the relationship between working memory capacity and the overall interpreting performance. Working memory capacity was found to support SI ability (Injoque-Ricle et al., 2015) and to be a strong predictor of SI performance (Macnamara and Conway, 2016). But in the study of Timarová et al. (2015), working memory capacity was only marginally significantly related to SI measures and only to such components with a predictable high memory component such as figures and lists of nouns. Generally, the correlation between working memory capacity and SI is found to be more common in the performance of untrained bilinguals and trainee interpreters than in professional interpreters. One explanation for this is that working memory capacity is thought to be a predictor at comparatively lower levels of skill acquisition and plays an essential role when the skill is still not yet automatic (Timarová et al., 2015). Studies relating cognitive abilities directly to fluency in interpreting have been scarce, but working memory capacity was found to predict SI fluency in trainee interpreters’ SI fluency performance in the study of Lin et al. (2018), which indicated the critical role of working memory capacity as compared with language skills in SI fluency.

As mentioned previously, although cognitive factors have been shown to have an essential role in the process of interpreting, the role of cognitive factors in interpreting still requires more empirical evidence to substantiate current findings. Utterance fluency, as a window to underlying cognitive processes and an important indicator of SI performance, provides a pertinent perspective to explore the cognitive bases of SI. This study proposes an exploration of fluency in L2 (English) to L1 (Chinese) SI performance of trainee interpreters. Given that the input rate of source speeches is an important influencing factor of cognitive load (Pöchhacker, 2004), this study takes input rate into consideration when examining the predicting power of cognitive fluency under conditions of low and high cognitive load, respectively. One of the important functions of formal training in interpreting and translation is to help trainees to enhance their performance to the full potential (Gile, 2009). The exploration into the predictive power of cognitive fluency into the development of SI utterance fluency development could shed light on the role of cognitive factors in interpreting expertise development. In addition, being a longitudinal study on the development of SI fluency, this research also has implications for the understanding of the information processing mechanism of interpreting and the development of interpreting expertise.

This study mainly aims to investigate the predicting power of cognitive fluency measures of trainee interpreters for their SI utterance fluency development under conditions of low and high cognitive load, respectively. The research questions were addressed through a series of multiple linear regression analyses. To this end, we obtained measures of cognitive fluency of trainee interpreters as predictors, including measures of lexical access, linguistic attention control, and working memory capacity, at the beginning of SI training. Measures of utterance fluency, the outcome measures for regression, were obtained through simulated SI tasks at the beginning and the end of an SI training period of 13 weeks.

## Materials and Methods

### Participants

Twenty-eight trainee interpreters of the master programs of interpreting from three universities in Hong Kong (26 female and 2 male participants) were recruited as participants in the study. They were all Chinese native speakers with English as the second language, except one participant who was a natural bilingual. Their mean age was 23.7 years old [standard deviation (*SD*) = 1.3]. Scores of IELTS (International English Language Testing System) was used as the index for general English proficiency (mean score = 7.4, *SD* = 0.4). The participants had on average received 1.6 years (*SD* = 0.2) of consecutive interpreting training and were all at the beginning stage of SI training by the time the experiments started. The training the participants received was comparable across the three universities. The participants received a 3-h SI classroom working session each week. The interpreting teachers provided participants with interpreting materials of real speeches and necessary background information for after-class practice in advance. During the classroom session, the teacher reviewed and gave feedback to the participants for their in-class interpreting of the practice speech. Instructions were given in terms of how to deal with difficulties encountered in the interpreting process, including possible SI strategies and skills that could be used such as anticipation, adjustment of ear-voice span, segmentation of message, and so on (Gile, 2009). The average SI practice time for participants was 18.1 h (*SD* = 1.2) each week during the period the experiment was conducted. Participants provided their written informed consent before the experiment and received cash reward for completing all sessions of experiments. All experimental procedures were approved by the Human Subjects Ethics Subcommittee of The Hong Kong Polytechnic University.

### Instruments

#### Predictor Measures: Cognitive Fluency Tasks

Three constructs of cognitive fluency of trainee interpreters were included in this study, that is lexical access, linguistic attention control, and working memory capacity. Correspondingly, three tasks administered were the semantic classification task, the category judgment task, and the speaking span task. The efficiency of the first two cognitive tasks was operationalized as the CV measures [coefficient of variance (CV)] of reaction time. Different from a change in reaction time due to the simple speed-up of processes, a change in the CV implies the restructuring of underlying cognitive processes (Segalowitz and Segalowitz, 1993). Coefficient of variance measures were calculated as an individual’s standard deviation of reaction time divided by his/her mean reaction time (Ankerstein, 2014). A lower CV reflects more stable reaction time after correcting for the overall responding speed and reflects more efficient processing (Segalowitz and Frenkiel-Fishman, 2005). Below are detailed descriptions of these tasks.

##### Lexical access: semantic classification task

The semantic classification task was adapted from the studies of Segalowitz and Freed (2004) and Segalowitz and Frenkiel-Fishman (2005). In this task, participants made speeded, two-alternative animacy judgment. Single nouns were presented on a computer screen, and participants were required to decide whether a word referred to an animate object or not through key responses on Chronos (Psychology Software Tools, 2020), an external device collecting key or sound responses with millisecond accuracy. The tests were conducted in both L1 (Chinese) and L2 (English). The English stimulus words were mostly translation equivalents of the Chinese stimuli. Pretests were conducted to ensure that all stimuli in both languages were familiar to bilinguals who had equivalent language competence to the participants. The frequency of stimuli was controlled, and all stimulus words were chosen from the list of the 5,000 most frequently used English and Chinese words or characters (Xiao et al., 2009; Davies and Gardner, 2010).

Both L1 and L2 versions of the task began with 30 practice trials, with 15 animate and 15 inanimate stimuli. Results of the practice trials were not included in the final analysis. The experimental procedure included the presentation of 50 animate and 50 inanimate words, recycled twice, leading to a total of 200 experimental trials. In each trial, the participant saw a fixation cross presented on the screen for 150 ms. Then, a stimulus was presented and would remain on screen for 3,000 ms until the participant made a key response on Chronos, followed by a blank screen for 500 ms. The order of stimuli was randomized. The order of task versions (L1 and L2) was counterbalanced across participants. Participants had a rest after 100 trials. Reaction time and accuracy were recorded.

##### Linguistic attention control: category judgment task

Linguistic attention control, measured through the category judgment task, was operationalized as shift cost, the ability to shift attention between two different attention-directing functions of words. Participants were required to perform the task in both L1 and L2 versions. Participants with better control of attention were supposed to make such shifts more efficiently.

The category judgment task, adapted based on previous research (Segalowitz and Frenkiel-Fishman, 2005), adopted the alternating runs paradigm (Rogers and Monsell, 1995). Two sets of stimulus words were used to explore the attention-directing function. One set of words referred to “the past” and “the future,” which directed the attention of participants to the temporal location of an event before the present moment (*ago*, *past*, *yesterday*, and *just now*) or after the present moment (*afterward*, *future*, *tomorrow*, and *soon*). The second set involved words of frequency, representing low frequency (*rarely*, *occasionally*, *seldom*, and *never*) or high frequency (*common*, *often*, *frequently*, and *always*). Participants were required to judge whether the presented stimulus words belonged to the past or the future for the time set of words, or the low or high frequency for the other set. Participants made key responses through Chronos.

Participants received instructions on how to make judgment of the time and frequency stimulus words before the task started, followed by four practice blocks of speeded classification trials. The eight time stimulus words in English were presented randomly at the center of the screen, recycled three times, leading to 24 trials in total for block 1. Block 2 was eight frequency stimulus words in the English version. Blocks 3 and 4 were the Chinese version of the time and frequency sets of stimulus words, respectively. Each practice block consisted of 24 trials. In each trial of the practice blocks, there was a fixation cross on the screen for 150 ms, followed by the stimulus word presented at the center of the screen. The stimulus would remain on the screen until the participants made a response or stay on the screen for 5,000 ms when there was no response. Participants were required to make judgments by pressing the response keys of Chronos as quickly as possible. After each block, there was a feedback of the error rate and mean reaction time of that block on the screen. Participants could choose to repeat the practice or to continue with the next block.

The tasks were administered in both L1 (Chinese) and L2 (English). Stimulus words of the Chinese version were mostly the translation equivalents of those in English. Eight L1 blocks and eight L2 blocks alternated, constituting 16 blocks in total. The order of the language of blocks was counterbalanced, for which half of the participants finished 16 blocks in the “L1L2L1L2…” order and the rest in “L2L1L2L1…” order. L1 and L2 blocks were distributed evenly across the session.

Within each block, the two judgment tasks—time and frequency—alternated. The time (T) and frequency (F) words were presented in the sequence “…TTFFTTFFTTFF…,” thus alternating between repeating and shifting conditions in a predictable way. Stimulus words were presented randomly, two adjacent words not being repeated. In each block, eight time words and eight frequency words were repeated three times, leading to a list of 48 stimuli. It has been shown in the previous research that subjects perform faster on repeat trials than on shift trials in alternating runs (Rogers and Monsell, 1995; Wylie and Allport, 2000; Monsell et al., 2003). The difference between repeating and shifting conditions is defined as shift cost, which reflects the extra burden the processing system carries in order to change the focus of attention (Segalowitz and Frenkiel-Fishman, 2005).

In each trial, stimuli appeared clockwise in the four quadrants of a square (10 cm × 10 cm) in the middle of the screen. Each stimulus word was presented at the center of one quadrant each time. The quadrants in which the first stimulus appeared were randomized across participants, an arrangement that meant that the first stimulus word might appear in any of the four quadrants. In the subsequent trial, a new stimulus, which moved clockwise around the screen, appeared in the adjacent quadrant of the previous one. Positions of a stimulus word served as visual cues as to which task (time or frequency judgment) was to be performed. In the experimental stage, each stimulus word would stay on the screen until the participant made response through Chronos keys or for 5,000 ms when there was no response. The response-stimulus interval was 150 ms. There was visual feedback for 20 ms when the response was incorrect. In case of an incorrect response, the stimulus–response interval was prolonged for an additional 1,500 ms to allow participants to recover. Data from the incorrect trials and the subsequent ones were discarded (Rogers and Monsell, 1995). The error rate and mean reaction time were presented on the screen after each block. There was a rest after every four blocks. The mean reaction time was registered, and CV measures of the repeating and shifting conditions in each language version were calculated. The shifting cost indexes in this study were calculated as CV measures under shifting conditions minus the corresponding measures under repeating conditions.

##### Working memory capacity: speaking span task

A speaking span task, a variant of the reading span task (Daneman and Carpenter, 1980), was conducted to test the working memory capacity of participants. The speaking span test taxes the processing and storage of memory simultaneously during the production process (Daneman and Green, 1986). Speaking span is found to be related to verbal fluency in both speech and reading tasks. Composite strict speaking span (CSSS) was calculated as the index for speaking span in this study, which takes three dimensions, that is, the processing accuracy, processing efficiency, and storage ability, into consideration and has been proven to better reflect the functions of working memory and predict utterance fluency (Jin, 2012). It was argued that the traditional measurement of speaking span might not reflect the differences in processing efficiency and storage ability (Weissheimer, 2007). Following the study of Jin (2011), processing accuracy is calculated as the number of syntactically and semantically acceptable sentences produced in the original form of presented words, not requiring the serial order of words; processing proficiency is the ratio of the time used to produce these sentences to the total number of sentences, reflecting the average reaction time; for the scoring processing proficiency, words recalled in the original order score 1 point each and otherwise 0.5 points each, and the average reaction time of correct responses is multiplied by −1; storage ability is the overall scores of words recalled, including those in incorrect sentences, derivative forms, or words recalled without formulating sentences. The CSSS is the average of the above three items after standardization.

The speaking span task in this research followed previous research (Christoffels et al., 2003; Jin, 2012). Sixty unrelated English (L2) words were selected. All stimulus words were high-frequency seven-letter words that were marked five points in terms of word frequency in the *Collins COBUILD Learner’s Dictionary*. The stimulus words were presented in the middle of the computer screen individually or 1,000 ms, followed by a 500-ms blank screen before the next stimulus appeared. The words were presented in three series, each of which contained 20 words. In each series, the two-word set was presented first, followed by the three-, four-, five-, and six-word sets consecutively. Words within each set were not related semantically or phonologically to prevent participants from memorizing the presented words. Participants were asked to read each word silently and remember the words. At the end of each set, a visual signal (question marks) appeared on the screen with an accompanying tone to signal the end of the set. The number of question marks represented the number of words in the set that was just presented. Participants were required to generate verbally a set of grammatically acceptable sentences (both semantically and syntactically) for each of the word just presented in the original order and form. There were no restrictions on the length and complexity of the produced sentences, or the position of the recalled word in the sentence. When participants had finished the recall and production of sentences of the current set, the next set of words was triggered until all 60 words had been presented. The tests were administered in both L1 and L2 versions because memory capacity might be different in native and second languages (Service et al., 2002).

#### Outcome Measures: SI Tasks

Simulated SI tasks that followed a 2 (training: pre/post) × 2 (input rate: low/high) factorial design were conducted. Participants interpreted two speeches from English (L2) to Chinese (L1), one with a high input rate and the other with a low input rate, simultaneously both at the beginning and end of an SI training period of 13 weeks. The four source speeches, two for pretraining tasks and two for posttraining tasks, were adapted from authentic speech videos. To ensure their comparability, all speeches were delivered by the same speaker, the Prime Minister of Singapore Lee Hsien Loong, for an annual event National Day Rally. The speeches were on general topics, with approximately 1,500 words for each speech. Efforts were made to ensure that the adapted speeches were linguistically comparable. A set of lexical, syntactic, and discourse parameters were derived to ascertain the comparability using Coh–Metrix (Graesser et al., 2004). The speeds of speeches were manipulated with Corel VideoStudio Pro X10 software to produce one slower speed (S) version (approximately 120 words per minute) and one faster speed (F) version (approximately 140 words per minute) for each speech. Two professional interpreters were invited to listen to the adapted speeches, and they confirmed that these speeches were natural for interpreters.

The SI tasks produced a total of approximately 225,000 Chinese characters in the interpreted output. A bilingual corpus of the participants’ interpreting output was built, with systematic annotations of indicators of utterance fluency with Elan 5.2 software (Wittenburg et al., 2006), which converted acoustic signals into an oscillogram and provided statistics of the frequency and duration of annotations. Source and interpreted speeches, silent and filled pauses, repairs, repetitions, and false starts in the speeches were annotated. The threshold duration of 0.3 s was adopted for an unfilled pause (Wang and Li, 2015). An annotation refers to one run of words between two unfilled pauses (≥0.3 s).

Measures of utterance fluency were selected based on previous studies on utterance fluency in language learning and interpreting (Mead, 2005; Kormos, 2006; Bosker et al., 2012; Han, 2015). The choice of utterance fluency indicators mainly followed the three dimensions of utterance fluency, that is, speed fluency, breakdown fluency, and repair fluency (Skehan, 2003; Tavakoli and Skehan, 2005). Speed fluency is relevant to speech rate (SR); breakdown fluency refers to hesitation phenomena of pauses, and repair fluency involves repairs, repetitions, false starts, and so on. This study chose representative indicators from these three dimensions and took features of fluency in interpreting into consideration. Speech rate, mean length of run (MLR), phonation time ratio (PTR), the mean number of silent pauses (SP mean), and the mean number of disfluencies (DF mean) were selected as indicators of SI utterance fluency. Pauses in interpreting reflect “highly directed, sometimes exclusive attention to input” (Setton, 1999, p. 246). Speech disfluencies reflect the increase in cognitive efforts demanded by lexical or syntactic uncertainty, planning, or production problems (Shreve et al., 2011). Disfluency mirrors difficulties and uncertainties during the cognitive processes of interpreting. In the current exploration, filled pauses were regarded as a type of disfluency, together with repairs, repetitions, and false starts following previous studies (Tissi, 2000; Cecot, 2001). Following Pöchhacker (1997), measures of SI utterance fluency indicators were adjusted to exclude extended pauses longer than 2 s in the source speeches to obtain a realistic indication of fluency measures in SI. Methods of calculation for indicators of utterance fluency are listed below:

SR: speech rate refers to the total number of words or characters produced, including disfluencies, divided by the total duration of speech (including pauses);
MLR: mean length of run is the number of words or characters in utterances between pauses of 0.3 s and above;
PTR: phonation time ratio is the percentage of the speaking time divided by the total time spent on producing the speech;
SP mean: the total number of silent pauses divided by the total amount of speaking time (adjusted for extended pauses >2 s in source speeches), expressed in seconds and multiplied by 60;
DF mean: the total number of disfluencies (filled pauses, repairs, repetitions, and false starts) divided by the total amount of speaking time (adjusted for extended pauses >2 s in source speeches), expressed in seconds and multiplied by 60.

### Procedure

Participants signed the consent form to participate in the experiment voluntarily and their personal data were collected, including demographic information, education background, interpreting experience, and IELTS scores. The four behavioral experiments were conducted at the beginning of the SI training. The order of L1 and L2 versions for each behavioral task was counterbalanced across participants. Participants made responses through Chronos and a microphone.

The simulated SI tasks were conducted in soundproof SI booths at the start and the end of an SI training period of 13 weeks. Participants interpreted two speeches simultaneously each time, and the four source speeches were linguistically comparable. Each participant interpreted a slower version of one speech and a faster version of the other for each SI task. The order of speeches and the order of speech versions (low or high input rate) were counterbalanced among the 28 participants by using Latin-square design. The SI tasks simulated real conference environment, and a small group of the audience listened to the interpreting on site. The speech videos were played on the screen of the computer in booths. Participants’ interpreting performance was recorded digitally with a double-track recording system.

For each SI task, participants familiarized themselves with the SI equipment before the experiment started. Each participant fulfilled the SI task in an individual booth. A briefing note of the topic of speech, background information of the speech and the speaker, and a glossary were distributed to participants in advance for their preparation. A warm-up speech made by the same speaker and on the same occasion allowed the participants to get familiar with the speaking style of the speaker. The participants only started the interpreting task until they were ready. After interpreting the first speech, participants filled a questionnaire to rate the level of difficulty of source speech and their SI performance in terms of content and fluency. Participants had a break for at least 10 min when finishing the first questionnaire to avoid the effects of fatigue. The procedure of interpreting the second speech was conducted in the same way as that of the first one. The posttraining SI task was administered at the end of the SI training period with the same procedure with that of the pretraining. Participants interpreted the other two speeches, which were made by the same speaker and were comparable to the two speeches used at the beginning of SI training.

## Data Analysis and Results

Multiple linear regression analyses were conducted to examine the predicting power of cognitive fluency for trainee interpreters’ SI utterance fluency development, under conditions of low and high input rates separately. Before regression analyses were conducted, descriptive statistics of cognitive fluency and utterance fluency development were presented. Correlation analyses between measures of cognitive fluency were conducted for a preliminary screening of predictors for the regression analyses. Correlation analyses between indicators of SI utterance fluency development were performed for a selection of representative dependent variables.

### Descriptive Statistics and Correlation Analyses

Descriptive statistics of cognitive fluency measures are summarized in Table 1. It presented the mean values, SD, and range of the RT (reaction time) and CV (coefficient of variance of RT) measures for the lexical access and linguistic attention control tasks, and the SSS (strict speaking span) and CSSS measures for the working memory span task. The current research used the CV measures of lexical access and linguistics attention control, and the CSSS measure of speaking span in its analyses as these indexes indicated the efficiency of cognitive fluency.

**TABLE 1:** Descriptive statistics of cognitive fluency parameters.

Cognitive fluency	Lexical access (LA)		Linguistic attention control (AC)		Working memory capacity (SS)	
	English	Chinese	English	Chinese	English	Chinese
	RT	RT	RT	RT	SSS	SSS
Mean	691.328	594.878	100.651	88.134	32.110	41.210
*SD*	96.262	87.869	98.503	132.205	8.257	6.191
Range	352.200	346.290	470.340	670.380	30.000	23.000

	**CV**	**CV**	**CV**	**CV**	**CSSS**	**CSSS**

Mean	0.278	0.273	0.032	−0.023	0.000	0.000
*SD*	0.076	0.084	0.107	0.093	0.738	0.688
Range	0.260	0.300	0.510	0.550	2.800	2.850

**RT, reaction time; CV, coefficient of variance of RT; SSS, strict speaking span; CSSS, composite strict speaking span*.*

To avoid the multicollinearity problem, correlation analyses were conducted to exclude predictors with significant correlations with other variables. Table 2 presents results of Pearson correlation analyses between cognitive fluency measures, including English lexical access (LA EN), Chinese lexical access (LA CH), English linguistic attention control (AC EN), Chinese linguistic attention control (AC CH), English speaking span (SS EN), and Chinese speaking span (SS CH). Results of analyses showed that LA EN was significantly related with LA CH (*r* = 0.804, *p* < 0.001). Significant correlations were also observed between SS EN and LA EN (*r* = 0.383, *p* < 0.05), LA CH (*r* = 0.547, *p* < 0.01), and SS CH (*r* = 0.491, *p* < 0.01). It was decided to exclude LA EN and SS EN, because of their significant correlations with other predictors. Measures of the remaining four parameters of cognitive fluency were retained as the independent variables for regression analyses, that is, Chinese lexical access (LA CH), English linguistic attention control (AC EN), Chinese linguistic attention control (AC CH), and Chinese speaking span (SS CH).

**TABLE 2:** Correlations of cognitive fluency parameters.

	LA EN	LA CH	AC EN	AC CH	SS EN	SS CH
LA EN	–					
LA CH	0.804**	–				
AC EN	–0.007	0.125	–			
AC CH	–0.32	–0.163	–0.066	–		
SS EN	0.383*	0.547**	0.296	–0.263	–	
SS CH	0.241	0.241	0.081	–0.26	0.491**	–

* Significant at the 0.05 level (two-tailed). ** Significant at the 0.01 level (two-tailed).

The descriptive statistics of SI utterance fluency measures under conditions of low and high input rates are summarized in Table 3. The mean value, SD, and range of SR, PTR, MLR, SP mean, and the DF mean in the pretraining and posttraining SI tasks were presented. Measures of SI utterance fluency development were indexed by partialing out correspondent measures of SI utterance fluency in the pretraining task from those in the posttraining task, and relevant statistics were also displayed in Table 3. Correlation analyses were conducted between indicators of SI utterance fluency development under conditions of low and high input rates separately, and the results are presented in Table 4. Under conditions of low input rate, SR was significantly related to PTR (*r* = 0.733, *p* < 0.01); significant correlations were also observed between MLR and SR (*r* = 0.624, *p* < 0.01), PTR (*r* = 0.583, *p* < 0.01), and SP mean (*r* = −0.496, *p* < 0.01). Under conditions of high input rate, SR was significantly related with PTR (*r* = 0.776, *p* < 0.01); significant correlations were also observed between MLR and SR (*r* = 0.648, *p* < 0.01), PTR (*r* = 0.724, *p* < 0.01), and SP mean (*r* = −0.782, *p* < 0.01). It was decided to exclude SR and MLR as they had significant correlations with other variables. Phonation time ratio, SP mean, and DF mean were retained as the dependent variables for regression analyses.

**TABLE 3:** Descriptive statistics of utterance fluency indicators.

		SR	PTR	MLR	SP mean	DF mean
**Low input rate**						
Pretraining	Mean	2.821	0.608	7.368	22.992	1.500
	*SD*	0.379	0.094	0.959	2.373	1.506
	Range	1.400	0.340	3.810	9.070	7.950
Posttraining	Mean	2.923	0.609	7.553	23.228	2.367
	*SD*	0.395	0.078	1.010	2.289	1.947
	Range	1.730	0.280	4.280	9.850	8.990
Utterance fluency development	Mean	0.102	0.001	0.185	0.236	0.868
	*SD*	0.263	0.054	0.767	2.030	1.165
	Range	0.880	0.210	2.920	7.260	6.600
**High input rate**						
Pretraining	Mean	2.968	0.610	7.908	22.560	3.049
	*SD*	0.424	0.092	1.139	2.551	2.718
	Range	1.630	0.350	5.000	10.590	14.600
Posttraining	Mean	3.060	0.614	8.103	22.695	2.371
	*SD*	0.385	0.088	1.061	2.444	2.390
	Range	1.680	0.290	4.580	10.470	12.770
Utterance fluency development	Mean	0.093	0.004	0.194	0.135	−0.679
	*SD*	0.231	0.054	0.898	2.201	1.146
	Range	1.100	0.230	4.120	9.490	4.680

**SR, speech rate (per second); PTR, phonation time ratio; MLR, mean length of run; SP mean, mean number of silent pauses per minute; DF mean, mean number of disfluencies (filled pauses, repairs, repetitions, and false starts) per minute*.*

**TABLE 4:** Correlations of measures of SI utterance fluency development.

	SR	PTR	MLR	SP mean	DF mean
**Low input rate**					
SR	–				
PTR	0.733**	–			
MLR	0.624**	0.583**	–		
SP mean	0.357	0.11	−0.496**	–	
DF mean	0.311	0.279	0.176	0.159	–
**High input rate**					
SR	–				
PTR	0.776**	–			
MLR	0.648**	0.724**	–		
SP mean	–0.061	–0.331	−0.782**	–	
DF mean	0.278	0.228	0.001	0.242	–

** Correlation is significant at the 0.01 level (two-tailed).

### Regression Analyses

To examine the predicting power of cognitive fluency for SI utterance fluency development, multiple linear regression analyses were performed, with LA CH, AC EN, AC CH, and SS CH as the predictors, and changes in PTR, SP mean, and DF mean as the dependent variables. All four predictors were entered into the regression as main effects, with the backward method. The regression analyses were performed for conditions of low and high input rates separately. Results of variables selection of backward regression analyses are presented in Table 5.

**TABLE 5:** Summary of variables selection results of backward multiple linear regression analyses.

Backward regression	Dependent variables	Variables removed	Variables entered
Low input rate	PTR	LA CH	AC EN *F*(1,27) = 3.453,
		AC CH	*p* = 0.074
		SS CH	
	
	SP mean	LA CH	
		AC EN	
		AC CH	
		SS CH	
	
	DF mean	AC CH	
		SS CH	
		LA CH	
		AC EN	

High input rate	PTR	AC EN	AC CH, SS CH *F*(2,27) = 6.655,
		LA CH	*p* = 0.005
	
	SP mean	AC EN	LA CH *F*(1,27) = 6.348,
		AC CH	*p* = 0.018
		SS CH	
	
	DF mean	LA CH	
		SS CH	
		AC EN	
		AC CH	

**Probability of F-to-remove ≥0.100 as the backward criterion*.*

Under conditions of low input rate, the predictor AC EN was retained in the model with PTR as the dependent variable. But the constructed model was not statistically significant, *F*(1,27) = 3.453, *p* = 0.074 > 0.05. The four predictors were all removed from the models with the dependent variables of SP mean and DF mean under low input rate conditions. It indicated that the explored cognitive fluency measures did not have a significant predicting power for changes in SI utterance fluency development (PTR, SP mean, and DF mean) under conditions of low input rate.

Under conditions of high input rate, the regression models with PTR [*F*(2,27) = 6.655, *p* = 0.005 < 0.01] and SP mean [*F*(1,27) = 6.348, *p* = 0.018 < 0.05] as the dependent variables reached statistical significance. The four predictors were all removed from the model with the dependent variable of DF mean under conditions of high input rate. Results of regression analyses for the dependent variables of PTR and SP mean under high input rate conditions are presented in Table 6.

**TABLE 6:** Results of backward regression models for PTR and SP mean under high input rate conditions.

Dependent variables	*R*^2^	Adjusted *R*^2^	Predictors	*B*	Std. error	β	*t*	*p*
PTR	0.347	0.295	(Constant)	0.011	0.009		1.23	0.23
			AC CH	0.292	0.097	0.503	3.009	0.006
			SS CH	0.037	0.013	0.464	2.774	0.01
SP mean	0.196	0.165	(Constant)	−3.048	1.319		−2.311	0.029
			LA CH	11.675	4.634	0.443	2.52	0.018

The regression model with PTR as the dependent variable accounted for 29.5% (adjusted *R*^2^) of the variance in the change in PTR, and the selected predictors were AC CH and SS CH. The other two predictors, AC EN and LA CH, were removed from the model. Results of *t*-tests for the regression coefficients showed that the efficiency of Chinese linguistic attention control (AC CH, *t* = 3.009, *p* = 0.006 < 0.01) and the efficiency of Chinese speaking span (SS CH, *t* = 2.774, *p* = 0.01 < 0.05) were significantly related to changes in PTR under high input rate conditions. AC CH and SS CH were positively related to the change in PTR with the coefficient 0.292 and 0.037, respectively.

The regression model with SP mean as the dependent variable accounted for 16.5% (adjusted *R*^2^) of the variance in changes in the SP mean, and the selected predictor was LA CH. The other three predictors, AC EN, AC CH, and SS CH, were removed from the model. Results of *t*-tests for the regression coefficients showed that the efficiency of Chinese lexical access (LA CH, *t* = 2.52, *p* = 0.018 < 0.05) was significantly related to changes in the SP mean under high input rate conditions. LA CH was positively related to the change in SP mean, with the coefficient of 11.675.

In order to further verify whether working memory capacity made an independent contribution in explaining the variance in PTR under high input rate conditions, hierarchical regression analyses were conducted. Results of hierarchical regression analysis with AC CH as the first block and SS CH as the second block of predictor showed that Chinese speaking span (SS CH) significantly increased the predicting power of the models (*Sig.*Δ*F* < 0.05). SS CH significantly enhanced the predicting power of the original model (with AC CH as the predictor) for changes in PTR (Δ*R*^2^ = 0.201, Δ*F* = 7.697, *Sig.*Δ*F* = 0.01 < 0.05) under conditions of high input rate, which implied that working memory capacity played an independent role in the overall predicting power of cognitive fluency. To examine whether SS EN and SS CH could contribute in the same way, another hierarchical regression analysis was conducted with AC CH as the first block and SS EN as the second block of predictor. The overall regression model was significant (adjusted *R*^2^ = 0.200, *p* = 0.024 < 0.05). Results of analysis showed that English speaking span (SS EN) did not significantly increase the predicting power of the models (Δ*R*^2^ = 0.112, Δ*F* = 3.790, sig. Δ*F* = 0.063 > 0.05). It implied that SS EN did not significantly enhance the predicting power of cognitive fluency for changes in PTR.

Multicollinearity, normality, and heteroscedasticity of the regression models were diagnosed to verify the reliability of the models. The VIF values of the independent variables were smaller than 2 (tolerance >0.5), indicating that the correlations between them were comparatively weak. Normal P–P plots of models indicated that residuals of the linear regression models obey normal distribution. Examination of the residuals’ scatterplots showed that the values of standardized residuals were all small and were distributed randomly, indicating there was no heteroscedasticity and verifying the reliability of the results of the linear regression models.

## Discussion

The primary goal of this study was to examine the predicting power of trainee interpreters’ cognitive fluency for their SI utterance fluency development under conditions of low and high cognitive load. To the best of our knowledge, this study is the first to investigate the relationship of cognitive fluency (with the three explored constructs) and utterance fluency development in SI. We conducted a longitudinal research by following 28 trainee interpreters at the MA level for an SI training period of 13 weeks. Different constructs of trainee interpreters’ cognitive fluency and dimensions of their SI utterance fluency were quantified. Results of regression analyses showed that trainee interpreters’ cognitive fluency could predict changes in some measures of utterance fluency in their SI output over a training period of 13 weeks. But the predicting power of cognitive fluency for SI utterance fluency development was only shown under high cognitive load. In this section, we discuss the overall predicting power of cognitive fluency and the role of individual constructs of cognitive fluency in the development of trainee interpreters’ SI utterance fluency. Directions for future work are also pointed out.

### The Predicting Power of Cognitive Fluency for SI Utterance Fluency Development

The predicting power of cognitive fluency for trainee interpreters’ SI utterance fluency development under high cognitive load conditions has been shown. Cognitive fluency measures could predict changes in the PTR and SP mean as indicators of SI utterance fluency development under conditions of high input rate. It indicates that the role of cognitive fluency in SI utterance fluency development is evident under cognitively high-demanding conditions. According to the embedded-processes model of working memory (Cowan, 2005), the capacity of the focus of attention is limited to three to five unrelated items, although chunking and structure can raise the limit. The activation of memory is extremely time-limited because of the severe time constraint in SI. With a high input rate, the interpreters have to process more messages in unit time, requiring more efficient cognitive processing. Higher efficiency in cognitive fluency makes more gains in utterance fluency possible under conditions of high cognitive load. It should be noted that the change in the ratio of phonation time as an indicator of SI utterance fluency development was small, although with a reasonable range, as shown in Table 3. This might be explained by the fact that trainee interpreters had to speed up the target language production while producing more messages after training under high time pressure, indicated by the increased SR and longer MLR. And a longer training period might bring a bigger change in PTR. Moreover, as SR, MLR, and PTR were significantly correlated, the overall predicting power of cognitive fluency for changes in speed fluency indicators (SR, MLR, and PTR) could be expected.

The results also showed that predictors for the development of SI utterance fluency in trainee interpreters’ output tended to be the efficiency of cognitive fluency in the target language. The significant predictors in the constructed models were measures of cognitive fluency in the target language, that is, AC CH and SS CH as predictors for changes in PTR, and LA CH as the predictor for changes in SP mean. SS CH significantly enhanced the predicting power for changes in PTR under high cognitive load, whereas SS EN did not. Because the language direction of the SI tasks was non-native to native language (English to Chinese), it implies that the efficiency of cognitive processes involved in the target language production stage, rather than that involved in the source language comprehension stage, tends to be predictors for the development of SI utterance fluency. This finding provides new evidence for the different demands of cognitive load in the comprehension and production processes of SI, which has implications for the information processing mechanism of interpreting. It is worth further exploration into different levels of cognitive load in SI and in the other language direction (native to non-native) to verify this finding in the future.

### The Role of Individual Constructs of Cognitive Fluency in SI Utterance Fluency Development

It is generally believed that SI requires the efficiency of lexical access in order to produce smooth delivery. Findings of this study showed that the efficiency of lexical access in the target language (LA CH) was significantly and positively related to changes in the SP mean under conditions of high input rate, but not under the condition of low input rate. It implied that there was a bigger change of the SP mean when the efficiency of lexical access was lower (bigger CV) under high input rate conditions. This finding confirms the correlation between the efficiency of lexical access and SI utterance fluency development. It is in line with Christoffels et al. (2003), who found a correlation between interpreting performance and lexical retrieval. But it should be noted that participants of the current study and those of Christoffels et al. (2003) were at comparatively lower level of interpreting expertise, trainee interpreters at the beginning stage, and untrained bilinguals. It is worth further exploration with professional interpreters as participants to verify this finding. Because English and Chinese lexical accesses were highly correlated, as reported in Section “Descriptive Statistics and Correlation Analyses,” similar predictive effects for changes in the SP mean could be expected from both. Future studies may consider using either of lexical access measures.

This study lends empirical support to the view that the shifting efficiency is a significant predictor of trainee interpreters’ SI utterance fluency development under conditions of high cognitive load. Linguistic attention control in this study differs from previous studies in which shifting is a function of domain-general cognitive control. The analytical results showed that the efficiency of linguistic attention control in the target language (AC CH) was significantly related to gains in PTR as indicators of SI utterance fluency development under high input rate conditions, but not under the condition of low input rate. The efficiency of linguistic attention control in the source language (AC EN) was not related to indicators of SI utterance fluency development significantly under either condition. This indicates that the efficiency of the target language processing seems to be main predictors for SI utterance fluency development. In addition, the findings of the present study generally support the view that the shifting function of cognitive control is important in interpreting performance (Timarová et al., 2014; Babcock and Vallesi, 2017). In future research, it is worth further investigation whether domain-general and domain-specific cognitive abilities make similar contribution to interpreting performance.

Our findings also provide evidence for the independent role of working memory in predicting the development of SI utterance fluency. Working memory capacity in the target language (SS CH) significantly increased the predicting power of cognitive fluency measures for changes in the PTR of trainee interpreters’ SI output under conditions of high cognitive load. This study confirmed the effectiveness of including working memory capacity in constructs of cognitive fluency in the investigation of SI utterance fluency development. It has implications for the inclusion of more constructs in the theoretical framework of cognitive fluency when applying it in the field of interpreting research. Working memory capacity in the source language (SS EN) did not significantly enhance the predicting power of cognitive fluency for PTR change as indicators of SI utterance fluency development under the same condition. This conforms with the domain-specific view of working memory in the sense that different cognitive resources are required for different domains of processing (Shah and Miyake, 1996; Miyake, 2001). It indicates that L1 and L2 working memory spans play different roles in interpreting performance, which is in line with findings of Cai et al. (2015). But our findings did not reveal a more important role of L2 working memory span in interpreting, which is different from the findings of previous studies (Christoffels et al., 2003; Cai et al., 2015). It might be due to the fact that the present study adopted the CSSS as the index for working memory capacity, which took the processing efficiency into consideration, whereas traditional measurement only paid attention to working memory capacity. The differences in tasks of memory span and modes of interpreting might be part of the reasons for the discrepancies. In addition, it is possible that L1 and L2 working memory resources play different roles in different constructs of the interpreting performance. The construct of fluency should be distinguished from the interpreting performance in content and fidelity and should be investigated separately in the future.

## Conclusion

The present study explored the predicting power of cognitive fluency for SI utterance fluency development of trainee interpreters by investigating the SI performance of 28 trainee interpreters over an SI training period of 13 weeks. Although changes in certain indicators of SI utterance fluency were limited during the tracked training period, the results provided evidence for the predicting power of cognitive fluency for SI utterance fluency development for the first time. It confirmed the effectiveness of the inclusion of working memory capacity in constructs of cognitive fluency in SI fluency research, and the roles of individual constructs of cognitive fluency in SI utterance fluency development were discussed. This study offers an interdisciplinary exploration of fluency in trainee interpreters’ SI performance, opening new perspectives of cognitive fluency research. The study has implications for the application of the theoretical framework of cognitive fluency in interpreting studies and bilingual language production research. It pays attention to the development of utterance fluency with a longitudinal approach and focuses on the efficiency of cognitive processes, providing methodological references for future relevant studies. Pedagogically, the identification of the role of cognitive fluency in SI fluency development sheds light on the possibility of including cognitive test in the interpreting aptitude test.

Several limitations of this research should be acknowledged. The limited size of participants and the gender imbalance may limit the generalizability of the findings of the current study. A larger and heterogeneous sample of participants, for instance, participants of different levels of interpreting expertise may be included in future studies. An adjustment of the results for multiple comparisons could possibly further enhance the statistical power of analyses. The present study only explored L2 to L1 SI performance. Future investigation into bidirectional SI may supplement existing findings. Besides, the training period of 13 weeks was comparatively short, which could partly account for the limited changes in certain indicators of SI utterance fluency. The tracking of a longer period of interpreting training might lead to more multifaceted findings.

## Data Availability Statement

The datasets generated for this study are available on request to the corresponding author.

## Ethics Statement

The studies involving human participants were reviewed and approved by the Human Subjects Ethics Sub-committee of The Hong Kong Polytechnic University. The patients/participants provided their written informed consent to participate in this study.

## Author Contributions

SS conceived and designed the experiments, performed the experiments, collected the data, performed the data analyses, and wrote the manuscript. DL revised the manuscript and contributed to the interpretation of results. Both authors approved the final version of the manuscript for submission.

## Conflict of Interest

The authors declare that the research was conducted in the absence of any commercial or financial relationships that could be construed as a potential conflict of interest.
